# Ignaz Philipp Semmelweis (1818–1865) – a public health visionary and champion of hand hygiene

**DOI:** 10.3205/dgkh000608

**Published:** 2025-12-11

**Authors:** Vanessa Ravel, Chandini Pandiyan, Krupa Chandran

**Affiliations:** 1 Independent Research Consultant, Chennai, India

**Keywords:** public health, hand hygiene, puerperal infection, historical vignette

## Abstract

As hygiene and infection control take center stage in modern healthcare, it is crucial to remember the struggle of a pioneer who championed these principles long before they were widely accepted. Ignaz Philipp Semmelweis is often regarded as the “Pioneer of Hand Hygiene,” the “Father of Infection Control”, and the “Protector of Motherhood" for his transformative approach in combating childbed fever. These titles only touch the surface of his broader legacy as a researcher and public health hero. Although he received limited recognition during his lifetime, Semmelweis’s posthumous legacy has laid the foundation for infection control and evidence-based medical practices. His enduring legacy is a powerful reminder of the importance of scientific inquiry and its lasting impact on public health.

## Abstract (de)

Da Hygiene und Infektionskontrolle im Mittelpunkt des modernen Gesundheitswesens stehen, ist es wichtig, sich an den Kampf eines Pioniers zu erinnern, der sich für diese Grundsätze einsetzte, lange bevor sie allgemein anerkannt wurden. Ignaz Philipp Semmelweis wird oft als „Pionier der Händehygiene“, als „Vater der Infektionskontrolle“ und als „Beschützer der Mutterschaft“ bezeichnet, weil er die Bekämpfung des Kindbettfiebers entscheidend vorantrieb. Diese Titel berühren nur die Oberfläche seines umfassenden Vermächtnisses als Forscher und Held der öffentlichen Gesundheit. Obwohl ihm zu Lebzeiten nur wenig Anerkennung zuteil wurde, hat Semmelweis' posthumes Vermächtnis den Grundstein für die Infektionskontrolle und für evidenzbasierte medizinische Praktiken gelegt. Sein bleibendes Vermächtnis ist eine eindringliche Erinnerung an die Bedeutung wissenschaftlicher Forschung und ihre nachhaltigen Auswirkungen auf die öffentliche Gesundheit.

## Background

**Figure 1:**
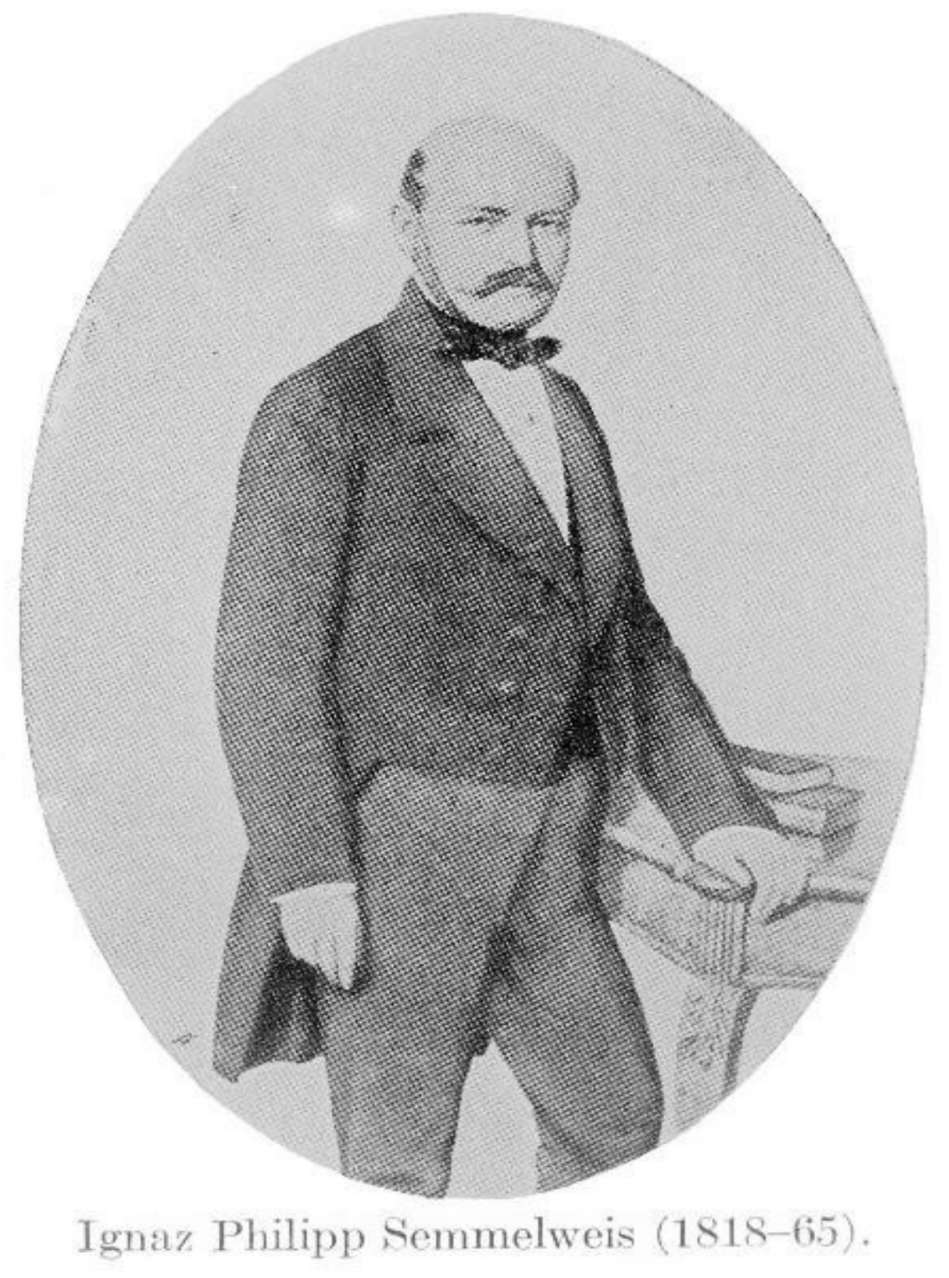
Ignaz Semmelweis (1818–65) (Permission obtained from Wellcome Collection)

Ignaz Philipp Semmelweis (1818–1865) was a key pioneer in the field of medicine, best known for his work on hand hygiene (Figure 1 (Fig. 1)). Popularly known as the “savior of motherhood” or the “conqueror of childbed fever”, Semmelweis worked relentlessly in investigating the cause of childbed fever [1]. Our article highlights his rigorous investigational prowess which revolutionized medical practices and contributed to modern public health strategies. Semmelweis was most likely the first physician during his era to use statistical methods to substantiate his findings, effectively advocating for this groundbreaking concept and demonstrating an unwavering commitment to evidence-based research [2], [3]. 

## Early foundations of a visionary thinker

Born on July 1, 1818, in the Taban district of what is now Budapest, Hungary. As a child, Semmelweis exhibited a keen intellect and profound curiosity, showing remarkable aptitude for many subjects. His early academic journey thus paved the way for a multidisciplinary future. 

Semmelweis first pursued liberal arts at the University of Pest for two years after completing high school. In 1837, following his father’s encouragement, Semmelweis enrolled himself at the University of Vienna to study law. However, the intricate world of human anatomy captivated him, leading him to abandon his law studies and shift to the medical program. He worked on his medical degree at the University of Pest, now known as Eötvös Loránd University, and graduated in 1844. Although he initially sought to specialize in internal medicine, circumstances did not favor this path, and he turned his focus towards obstetrics. He began his master’s training at the Vienna General Hospital in Austria. On July 1, 1846, at the age of 28, he became an assistant to Johann Klein, a physician in the maternity division at the same hospital [4], [5], [6]. His responsibilities included examining patients every morning to prepare for the professor's rounds, managing complicated deliveries, educating obstetrics students, and serving as the ‘clerk’ of records [7].

## From miasma to evidence- based practices

**Figure 2:**
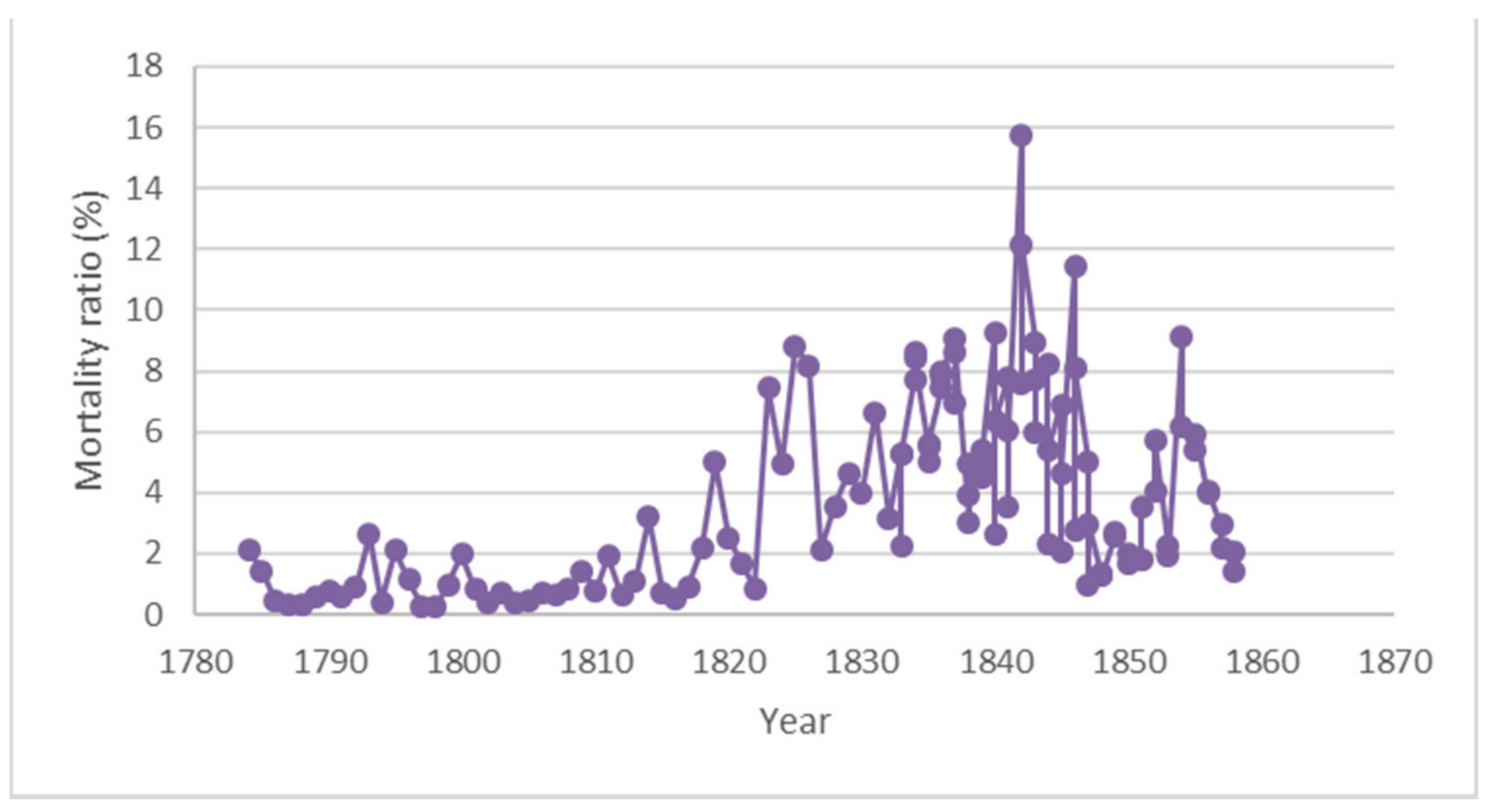
Annual mortality rates at the Vienna hospital from 1784–1858 (graph constructed by the authors based on Semmelweis’s statistics as mentioned in his publication [16], [18])

Puerperal fever, also known as childbed fever, was an epidemic that claimed the lives of innumerable mothers in Europe and America during the early to mid-19th century. This disease typically impacted women during the first three days following childbirth. The years 1760–1850 marked an important phase with advancements in the understanding of puerperal fever with emerging theories on the cause and spread [8]. The following dramatic situation was typical of the time: “A woman could be delivered on Monday, happy and well with her newborn baby on Tuesday, feverish and ill by Wednesday evening, delirious and in agony with peritonitis on Thursday, and dead on Friday or Saturday” [9]. The annual mortality rates from 1758–1854 at the Vienna hospital are outlined below (Figure 2 (Fig. 2)). 

Bloodletting and extensive purging was the treatment of choice during that time and it proved to be largely ineffective [7], [8], [10]. Until the late 1800s, the concept that disease may be caused by specific microorganisms had not been established. The germ theory had not yet emerged, leaving the prevention and treatment of these diseases without a solid scientific foundation [11]. Prevailing theories attributed infections to “bad air” or “miasmas” and also mentioned that it could be an imbalance of bodily humors. It was during this period that the efforts of two prominent medical practitioners, Oliver Wendell Holmes and Ignaz Philipp Semmelweis, became crucial. Their research challenged the prevailing theories by demonstrating the role of infection in puerperal fever, which contradicted the then-accepted explanations of disease causation [10]. 

Semmelweis’ efforts spared the lives of countless women and this pivotal time in the history of medicine came to be known as the “golden age of the physician scientist” [12]. During this period, there was a significant shift from outdated beliefs to a focus on research and data collection. Physicians showed a growing interest in numbers and empirical data [13]. Semmelweis’s commitment marked him as one of the few scientists who pursued a more rigorous, evidence-based approach to decipher the underlying cause of puerperal fever. 

## Decoding the mystery: Semmelweis’s investigations and interventions

The Allgemeine Krankenhaus Vienna Hospital had two obstetrics clinics [14]. The first clinic was opened in 1784. In 1833, the second clinic opened. From 1840, medical students and junior physicians were taught in the first clinic, and midwives in the second clinic. By the 1850s and 1860s, the hospital treated about 7,000 to 8,000 patients a year. In July 1846, Semmelweis was appointed to oversee the First Obstetrical Clinic. It was then that he noticed that the deaths due to puerperal fever in the first clinic was over twice that of the second [15].

Semmelweis sought to understand the reason why the first clinic had a significantly higher incidence of puerperal fever than did the second clinic. In an era when physicians strongly believed that causative factors for a disease were either due to vague environmental factors or imbalance in bodily humors, he embarked on systematic inquiry. As a critical first step, he meticulously recorded and compared mortality rates across different wards. By systematically analyzing this data, he tried to ascertain potential explanations for the observed disparities [2], [15], [16]. 

First, he scrutinized obvious reasons like birthing positions. In the second clinic, pregnant women delivered while lying on their sides, while in the first clinic, they gave birth lying on their backs. Semmelweis hypothesized that this difference might be the reason, but changing the birthing position in the doctors’ ward (the first clinic) had no effect on the mortality rate. 

Next, Semmelweis observed that whenever a patient died of childbed fever, a priest would perform a ritual where he would walk around the clinics ringing a bell. He had to walk through five wards before reaching a dying woman's room. In contrast, the priest at the second clinic had direct access to the sickroom. He theorized that this ritual induced fear in the patients, potentially leading to fever and death. He asked the priest to change his route and stop the practice of ringing the bell, but the adjustment also failed to influence the death rates in the clinic. 

He ruled out “overcrowding” as a cause, noting that mortality rate was lower in the overcrowded second clinic compared to the first clinic. He also ruled out climate as a contributing factor as it remained consistent in both clinics [15], [16], [17]. His approaches received severe backlash from the medical community. Semmelweis’s persistence and scientific rigor set him apart from his contemporaries. His work was not just experimental – it was the early foundation of epidemiology and infection control. 

## The great breakthrough

Frustrated by the repercussions and in dire need of a new perspective, Semmelweis stepped away from his hospital responsibilities and journeyed to Venice, hoping that this change might offer new insights. Upon his return, he encountered a tragic event: his pathologist colleague, Jakob Kolletschka, had died from childbed fever after accidentally pricking his finger during autopsy of a woman who died from the same illness. Semmelweis hence theorized that puerperal fever was not limited to postpartum women but could also affect others within the hospital environment. 

He then sought to compare the clinical practices in the two clinics and found that the doctors and students in the first clinic frequently performed autopsies, while the midwives in the second clinic were only involved in birthing. He therefore hypothesized that the cadaverous particles might have been transferred to women by the physicians during childbirth, leading to infection and fever. 

**Figure 3:**
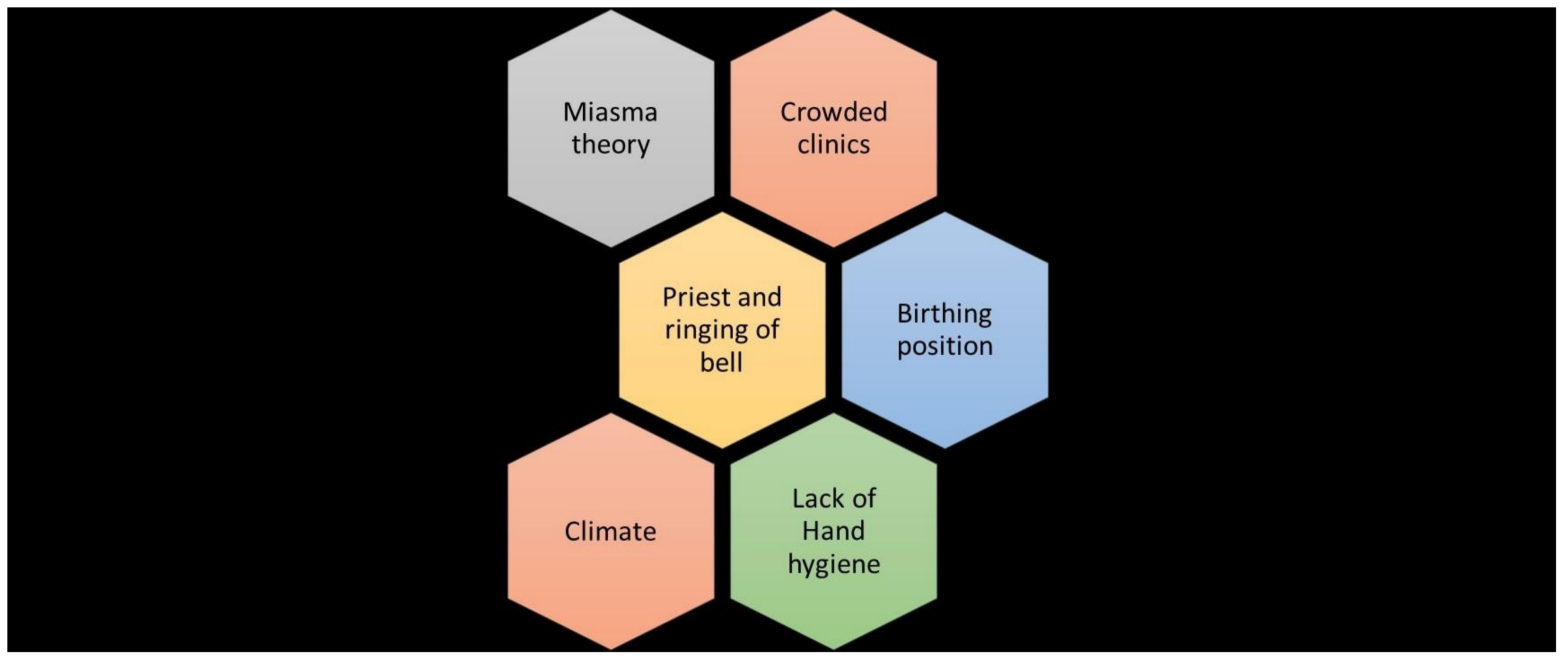
Exploration of Semmelweis’s hypotheses for the causative factor of puerperal fever (image prepared by the authors based on the Semmelweis publication [16], [18])

This breakthrough theory famously termed “unholy” hands of “holy” physicians highlighted the role that hygiene plays in the transmission of puerperal fever (Figure 3 (Fig. 3)) [1], [16], [17], [18]. In 1858, Semmelweis described his findings in two papers titled "The Aetiology of Childbed Fever" and "The Difference in Opinion between Myself and the English Physicians regarding Childbed Fever” [2]. After a long wait, he successfully published his book “Die Ätiologie, der Begriff und die Prophylaxis des Kindbettfiebers” (1861) (The Etiology, Concept, and Prophylaxis of Childbed Fever) [16], [18]. 

## The hand washing protocol: A pioneering method with enduring impact

**Figure 4:**
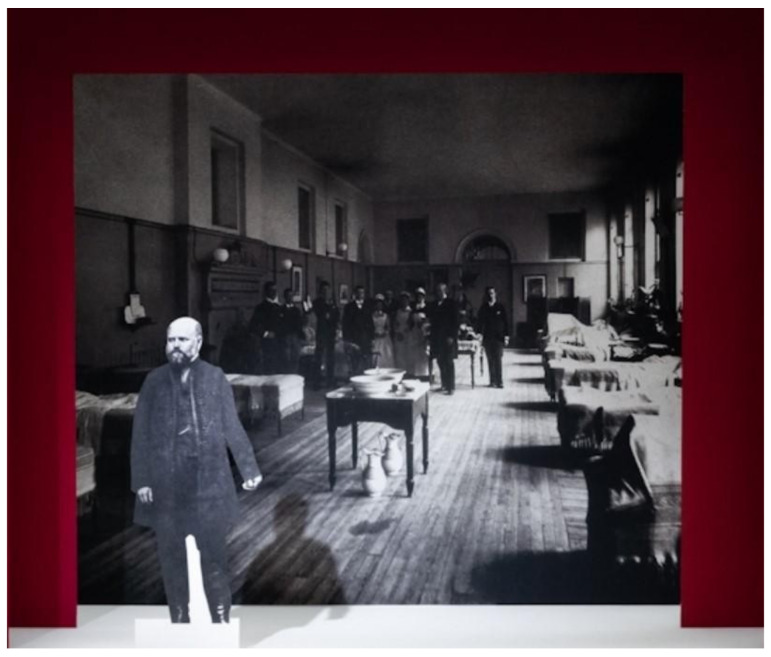
Semmelweis placed chlorine solution at the entrance of the first clinic, requiring every medical attendant to wash their hands before touching a woman in labour (permission obtained from Wellcome Collection; Photographer: Steven Pocock)

Unlike his contemporaries, who either focused on population level interventions (John Snow) or broader epidemiological theories (Pasteur) or vaccine development (Jenner), he focused more on individual-level intervention and a practical, hands-on solution. He approached the problem with the precision of a modern researcher, effectively executing one of the earliest trials in infection control. Semmelweis introduced a hand-washing protocol using chlorine solution (chloral lime), renowned for its powerful disinfectant properties. He mandated that the physicians in the first clinic wash their hands and clean under their fingernails after performing autopsies and before interacting with patients (Figure 4 (Fig. 4)) [17].

**Figure 5:**
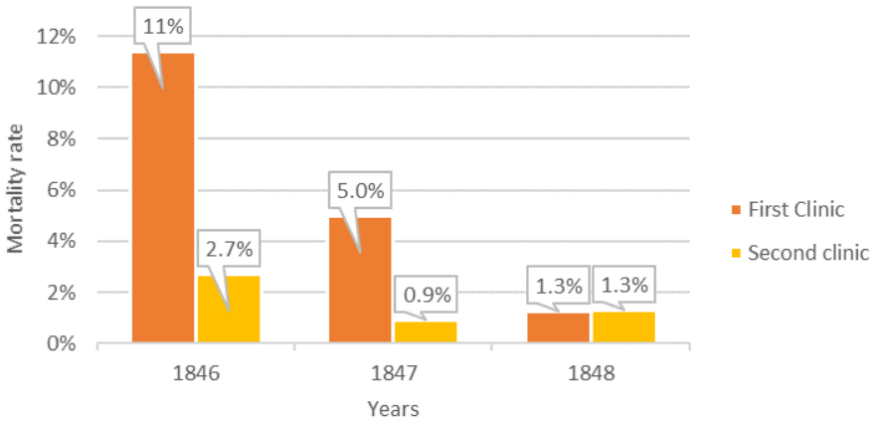
Maternal mortality rates for the first clinic and second clinic (1846–1848). The rates dropped markedly when Semmelweis implemented hand hygiene mid-May 1847 (image prepared by the authors based on the Semmelweis publication [16], [18])

As a result, the first clinic’s mortality rate dropped by 90% and became nearly equivalent to that of the second one. In 1846, out of 4,010 patients in the first clinic, 459 patients died (11.4%) and in the second clinic out of 3,754 patients, 105 patients (2.7%) died before the implementation of chlorine washing. In 1847, after implementation of chlorine washing around Mid-May, out of 3,490 patients, around 176 died (5%) and in the second clinic around 32 patients (0.9%) died. In 1848, hand hygiene was implemented consistently throughout the year, and out of 3,556 patients, 45 (1.3%) died in the first clinic. In the second clinic of the same year, 43 out of 3,219 patients died, which was about 1.3% (Figure 5 (Fig. 5)) [16], [17], [19]. Semmelweis conducted what could be compared to a randomized controlled trial on hand hygiene, the first of its kind, which resulted in dramatically reduced maternal mortality rates [2], [17]. He had also demonstrated his innovative thinking as a researcher through his early preclinical experiments on nine rabbits in 1849 [5], [6], [20].

Semmelweis’s groundbreaking discovery underscored the vital role of hand hygiene in preventing the spread of infectious diseases – a principle that remains foundational in public health even today. This transformative approach established him as the champion of health promotion and showcased his commitment to patient safety and quality of care, which are the fundamental responsibilities of every physician to ensure high standards of medical care. 

Since then, hand washing has saved millions of lives. From the 1980s, Semmelweis’s principles have been institutionalized, marking a landmark in the evolution of hand hygiene in healthcare [21], [22]. By the early 2000s, alcohol-based hand rubbing was recognized as the preferred method of maintaining hygiene in medical environments, further solidifying the lasting impact of Semmelweis’s discovery [23], [24]. His concept laid the foundation for the World Health Organization’s (WHO) recommendations on hand hygiene, ‘When to hand rub’ and the ‘5 Moments for Hand Hygiene’ [25], [26], [27], [28].

The relevance of Semmelweis’s work, which transformed medical practices in his time, continues to do so even today. “The COVID-19 pandemic is a strong reminder that one of the most important, simplest and cost-effective ways to reduce the current coronavirus outbreak is hand hygiene” [29].

## Overlooked advocacy: Semmelweis’s struggle in advancing hand hygiene

Despite Semmelweis’s success in significantly reducing mortality rates, his groundbreaking work encountered considerable opposition from colleagues. His insistence on strict hand hygiene practices met with reluctance, especially from obstetricians who favored their own theories over Semmelweis's findings, even though the cause had been proven through intervention. Prominent persons such as Charles Delucena Meigs, a highly influential obstetrician at the time, rejected the idea of contagion, famously questioning why such a “virus” would only affect women who had recently given birth and no one else. 

“How come then, that a mortal virus or contagion should have power over a woman who is pregnant, or recently delivered, while it is innocuous for all others in the world?” - Statement by the physician Charles Delucena Meigs [5].

**Figure 6:**
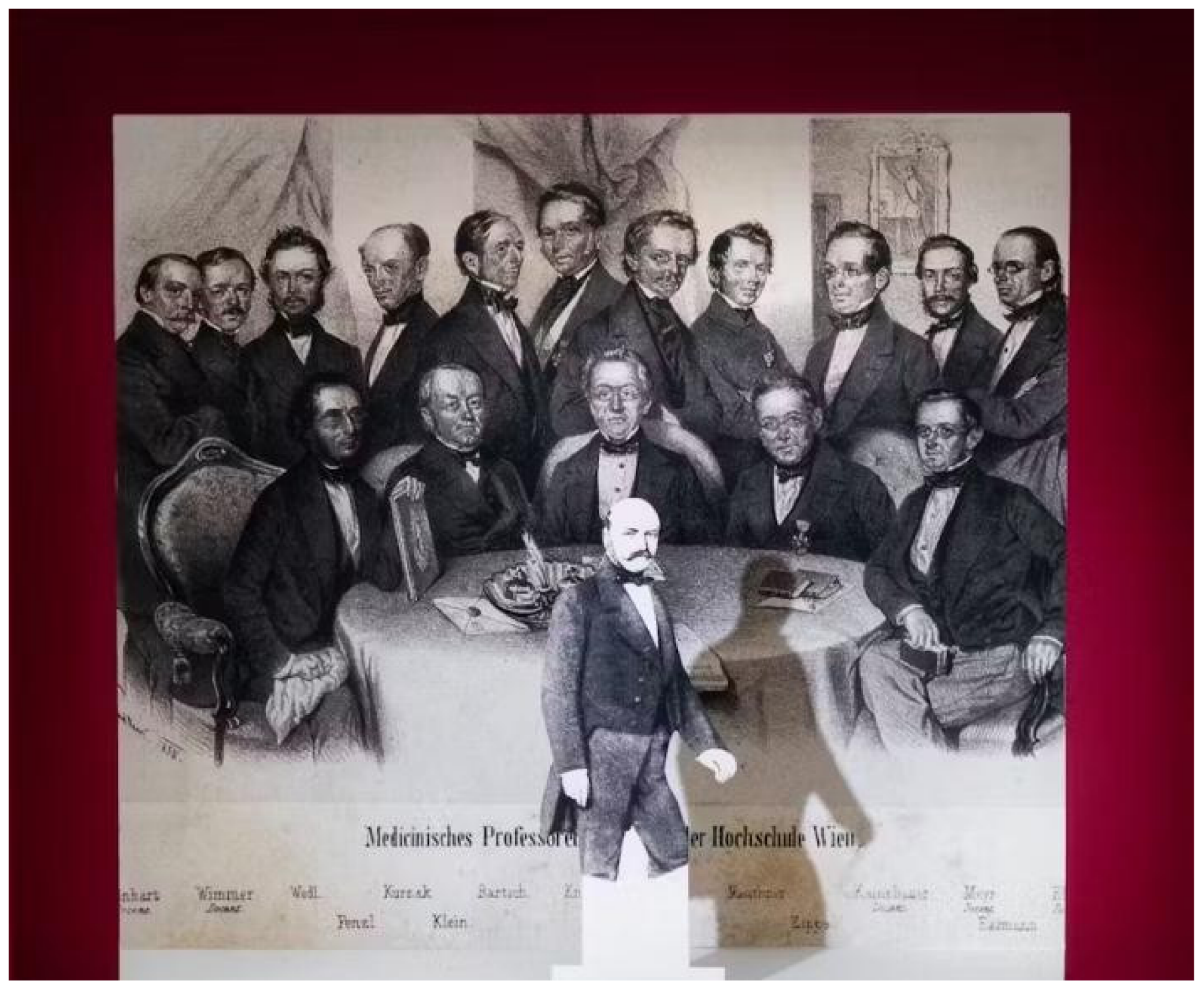
Semmelweis the lonely man trying to combat childbed fever fighting against resistance imposed by obstetricians (permission obtained from Wellcome Collection. Photographer: Steven Pocock)

Others, like Ede Flórián Birly, believed that puerperal fever stemmed from bowel infections, while Carl Braun attributed it to miasma. Many simply refused to believe that a single intervention, such as chlorine handwashing, could be the solution. Semmelweis was unable to obtain a “Privatdozent” title for his groundbreaking discovery and was eventually reduced to a theoretical professor position [5], [30]. (Fig. 6)

One reason for this resistance could be that Semmelweis’s theory suggested that doctors themselves were responsible for spreading puerperal fever, inadvertently transferring decaying matter to patients during examinations. This implication was not well received by the medical community, as it cast a harsh light on their practices. Even within Vienna, where Semmelweis worked, his ideas lost favor, with figures like Friedrich Wilhelm Johann Ignaz Scanzoni and Bernhard Seyfert manipulating mortality data to argue against the effectiveness of chlorine handwashing [5].

In addition to external opposition, Semmelweis’s own actions contributed to the slow acceptance of his ideas. Despite his excellent skills in practical work and investigations, he harbored an “innate aversion” to writing, as he noted in the preface of his 1861 publication [17]. His findings were initially reported not by him, but by his friend Ferdinand von Hebra in a December 1847 article, followed by another in April 1848. Semmelweis was invited to present his findings to the Vienna Medical Society in 1849, but he declined the opportunity, leaving his colleague Josef Skoda to speak on his behalf [6], [17]. This reluctance to personally share and publish his work further hindered its widespread acceptance. 

## Unrecognized heroism – the one-man army

“The medical literature for the last twelve years continues to swell with reports of puerperal epidemics…. In published medical works, my teachings are either ignored or attacked. The medical faculty at Würzburg awarded a prize to a monograph written in 1859 in which my teachings were rejected…” – words from a frustrated visionary whose insights were dismissed [7], [16] (Figure 6 (Fig. 6)). 

This quote expresses his frustration as he saw his ideas rejected by the leading obstetricians of the time. Regardless of an abundance of evidence supporting his results, his theories faced strong resistance from strong medical traditions as well as deeply held opinions about causality of diseases. He found himself isolated, which was due to an absence of communication channels and peer support networks. Semmelweis’s experiences are somewhat akin to the challenges that public health researchers face while advocating for evidence-based practices in the face of cultural myths and resistance. As we explore complicated health problems in a rapidly changing medical landscape, it is critical to create an environment that welcomes and supports innovative ideas. 

He was committed to a lunatic asylum in 1865, where he succumbed to sepsis on August 13^th^ at the age of 47 years. Semmelweis's tragic fate as an unsung hero of medicine has left an irrevocable impression in the hearts of many researchers. His pioneering work in hand hygiene, though initially overlooked, was a monumental leap forward in patient safety and quality of care. Many biographers of Semmelweis concur that his tragic life experience deteriorated his mental health ultimately making him a “martyr to the world's stupidity” [3], [31]. 

## Honoring the legacy

The Semmelweis University for Medicine and Health-related disciplines in Budapest, the Semmelweis Klinik in Vienna and the Semmelweis Hospital in Miskolc are a few amongst many honored establishments that have been named after him. His Budapest home is now known as the Semmelweis Medical History Museum [7]. 

## Lessons from Semmelweis’s public health journey

Semmelweis’s legacy serves as a testament to the power of innovative research in public health. In an era when his ideas were questioned, Semmelweis demonstrated outstanding courage and a commitment to improving maternal and infant health outcomes. His story serves as an essential reminder that we must embrace pioneering ideas that challenge established norms rather than succumbing to the Semmelweis reflex – a natural inclination to reject groundbreaking ideas. As we remember Semmelweis's unwavering commitment to advancing public health, we must also work to foster a culture of inquiry and acceptance. By doing so, future medical and public health professionals can drive health promotion efforts forward, ensuring that his vision for safer, healthier communities lives on. 

## Notes

### Authors’ ORCIDs

- Vanessa Ravel: 0000-0001-6740-5481
- Chandini Pandiyan: 0009-0007-5405-7933
- Krupa Chandran: 0000-0002-8344-3549

### Funding

None. 

### Competing interests

The authors declare that they have no competing interests.
